# Disease Rescue and Increased Lifespan in a Model of Cardiomyopathy and Muscular Dystrophy by Combined AAV Treatments

**DOI:** 10.1371/journal.pone.0005051

**Published:** 2009-03-31

**Authors:** Carmen Vitiello, Stefania Faraso, Nicolina Cristina Sorrentino, Giovanni Di Salvo, Edoardo Nusco, Gerardo Nigro, Luisa Cutillo, Raffaele Calabrò, Alberto Auricchio, Vincenzo Nigro

**Affiliations:** 1 Telethon Institute of Genetics and Medicine (TIGEM), Naples, Italy; 2 Dip. di Scienze Cardiotoraciche e Respiratorie, A.O. Monaldi, Seconda Università degli Studi di Napoli, Naples, Italy; 3 Genetica Medica, Dip. di Pediatria, Università Federico II, Naples, Italy; 4 Laboratorio di genetica medica, Dip. di Patologia Generale, Seconda Università degli Studi di Napoli, Naples, Italy; University of Florida, United States of America

## Abstract

**Background:**

The BIO14.6 hamster is an excellent animal model for inherited cardiomyopathy, because of its lethal and well-documented course, due to a spontaneous deletion of delta-sarcoglycan gene promoter and first exon. The muscle disease is progressive and average lifespan is 11 months, because heart slowly dilates towards heart failure.

**Methodology/Principal Findings:**

Based on the ability of adeno-associated viral (AAV) vectors to transduce heart together with skeletal muscle following systemic administration, we delivered human delta-sarcoglycan cDNA into male BIO14.6 hamsters by testing different ages of injection, routes of administration and AAV serotypes. Body-wide restoration of delta-SG expression was associated with functional reconstitution of the sarcoglycan complex and with significant lowering of centralized nuclei and fibrosis in skeletal muscle. Motor ability and cardiac functions were completely rescued. However, BIO14.6 hamsters having less than 70% of fibers recovering sarcoglycan developed cardiomyopathy, even if the total rescued protein was normal. When we used serotype 2/8 in combination with serotype 2/1, lifespan was extended up to 22 months with sustained heart function improvement.

**Conclusions/Significance:**

Our data support multiple systemic administrations of AAV as a general therapeutic strategy for clinical trials in cardiomyopathies and muscle disorders.

## Introduction

Limb-girdle muscular dystrophies 2C-2D-2E-2F (LGMD2C–2F) are classified as “sarcoglycanopathies”. They have typically a childhood onset and represent the most severe forms of LGMD, often associated with cardiomyopathy [1]. Alpha-, beta-, gamma-, and delta-sarcoglycan are arranged at the muscle membrane in the form of a heterotetrameric dystrophin-associated complex at a 1∶1∶1∶1 ratio. When one component is absent, the other sarcoglycans are displaced and degraded, since the stability of the complex is impaired. Sarcoglycan complex is also secondarily reduced in Duchenne muscular dystrophy (DMD), when dystrophin is missing [2], indicating that DMD muscle suffers from combined dystrophin and sarcoglycan complex deficiencies [3]. The BIO14.6 hamster displays absence of delta-sarcoglycan from the muscle membrane, followed by the deficiency of alpha, beta and gamma sarcoglycan, reproducing the human LGMD2F phenotype [4]. This animal model was generated in 1962, when Homburger fixed by repeated inbreeding a spontaneous trait of Syrian hamster characterized by muscular dystrophy and cardiomyopathy [5]. All the other cardiomyopathic hamsters are derived from this first strain, among which TO-2, UM-X7.1, presenting different types of cardiomyopathy. All strains carry the same homozygous 24-kb deletion of the delta-sarcoglycan (δ-SG) gene promoter and first exon [6]. Interestingly, Zhu et al. used for gene therapy the TO-2 hamster strain that show a shortened lifespan caused by rapid congestive cardiomyopathy without hypertrophy, with minimal muscle disease [7]. This study was completed after 48 weeks and single AAV treatment was effective. We chose to study the efficacy of gene therapy on the original BIO14.6 hamster, since it is the best characterized strain with over 1,000 citations, to date. Disease stages are uniform within BIO14.6 strain due to the homogeneous genetic background. Differently from the dystrophin-deficient *mdx* mouse that shows muscle regenerative capacity and lives 80% of wild type (WT) mice [8], the average lifespan of BIO14.6 hamsters is 11 months (about 40% of the WT hamster), because the heart dilation slowly progresses to an ejection fraction of 20–30%, leading to heart failure. In addition, the BIO14.6 hamster displays a continuous loss of muscle strength in the course of the life and has no successful muscle regeneration. The heart pathology observed in this animal model is similar to human X-linked cardiomyopathy, in which the heart dystrophin is absent [9].

Adeno-Associated Viruses (AAVs) are non-pathogenic single-stranded DNA parvovirus. Each end of the single-stranded DNA genome contains an inverted terminal repeat that is mandatory for replication and packaging. The promoter and transgene cDNA are inserted between the two inverted terminal repeats, with Rep and Cap provided in trans. Because there are no viral genes in the recombinant AAV, vector toxicity should be avoided. All the AAV used for gene therapy studies are transcapsidated: that is to package an AAV genome containing an ITR from one serotype (AAV2) into the capsid of another serotype (i.e. AAV 2/1): this allows expanding the repertoire of AAV for better targeting of cell types [10, and references therein]. AAV1 and AAV6 are very similar with six amino acid differences, AAV8 was isolated from rhesus monkey, while AAV9 from human tissues. AAV-mediated gene transfer to muscle demonstrated long-term persistence. In hemophilia B dogs, factor IX (FIX) was recovered >4 years following AAV2-FIX injection in both muscle biopsies and circulation. Haemophilia B patients receiving intramuscular injections of AAV encoding FIX were shown to have consistent local expression of FIX up to 3.7 years following delivery [11]. Interestingly, systemic administration of AAV 2/1, 2/6, 2/8 or 2/9 resulted in efficient body-wide muscle transduction. This can be suitable for treating genetic disorders affecting all muscles [12]–[14]. We used male BIO14.6 hamsters to test AAV serotypes 2/1, 2/8 and 2/9. We compared different protocols of systemic gene delivery and monitored the progression of heart and muscle disease for each group of injected animals. We observed a general improvement in each group of treated animals, in particular in the hamsters injected at 2 weeks with serotype 2/8. To test long-term transgene expression, we also used double AAV injections with two different serotypes to elude immune reaction. Hamsters received the serotype 2/8 at 2 weeks and 2/1 at 5 months of age. We observed a normal lifespan in the double injected hamsters, due to persistent rescue of skeletal muscle and heart structure and function.

## Results

**Figure 1:**
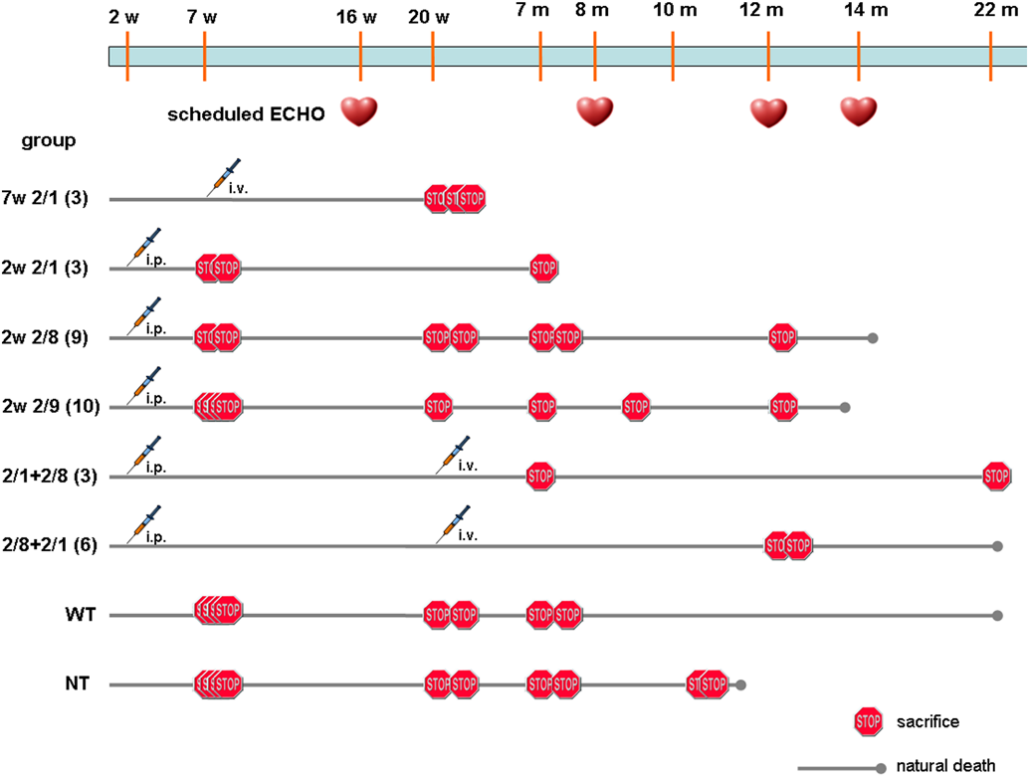
Timelines of the experimental procedures for the different groups of hamsters. w: weeks; m: months; WT: Wild-type hamsters; NT: BIO14.6 hamsters not treated; i.p. : intraperitoneally; i.v. : intravenously; 7w 2/1: hamsters injected at 7 weeks of age with δ-SG by AAV 2/1; 2w 2/1, 2w 2/8, 2w 2/9: hamsters injected at 2 weeks by AAV 2/1, 2/8 and 2/9, respectively; 2/1+2/8: hamsters injected at 2 weeks by AAV 2/1 and at 5 months with δ-SG using AAV 2/8; 2/8+2/1: hamsters injected at 2 weeks by AAV 2/8 and at 5 months using serotype 2/1. The timeline stops when all the hamsters of the group are dead.

To compare the efficiency of alternative protocols for systemic gene delivery into hamsters, we measured the following parameters at different times after the treatment: i) degree and distribution of human δ-SG expression; ii) expression of the other components of the sarcoglycan complex; iii) muscle pathology; iv) cardiac and skeletal muscle function (Figure 1). All animals were male.

### Protocol design

The parameters varied in the alternative protocols were: 1) timing of delivery; 2) AAV serotypes; 3) number of injections; 4) route of injection. The AAV dose was kept at 4×10^∧^13 GC/kg for all the experiments.

Figure 1 shows the experimental timelines of six different groups of hamsters.

Group 7w 2/1: we injected AAV2/1-CMV-hSCGD into three 7-week-old (approximately 60 g) BIO14.6 hamsters through the jugular vein.

Group 2w 2/1: it comprises three BIO14.6 hamsters intraperitoneally treated at two weeks of age (approximately 15 g) using AAV2/1-CMV-hSCGD.

Group 2w 2/8: it includes nine BIO14.6 hamsters intraperitoneally treated at two weeks of age with AAV2/8-CMV-hSCGD.

Group 2w 2/9: it consists of ten BIO14.6 hamsters intraperitoneally treated at two weeks of age with AAV2/9-CMV-hSCGD.

Group 2/1+2/8: it includes three hamsters intraperitoneally injected at 2 weeks of age with AAV2/1-CMV-hSCGD and then, at 5 months of age, with AAV2/8-CMV-hSCGD via the jugular vein.

Group 2/8+2/1: it includes six hamsters intraperitoneally injected at 2 weeks of age with AAV2/8-CMV-hSCGD and then, at 5 months of age, with AAV2/1-CMV-hSCGD via the jugular vein.

The AAV serotype of the second injection was always changed to avoid any anti-capsid immune response. We verified and excluded the presence of neutralizing antibodies against AAV2/1 or AAV2/8, in groups 2/1+2/8 and 2/8+2/1 respectively, prior to the second AAV administration. As expected, we did not observe any response against δ-SG that is expressed at about 3% in BIO14.6 hamsters, since an alternative upstream promoter is activated. On the other hand, sequence conservation between human and hamster δ-SG is 98%.

### Expression of the SG complex

**Figure 2:**
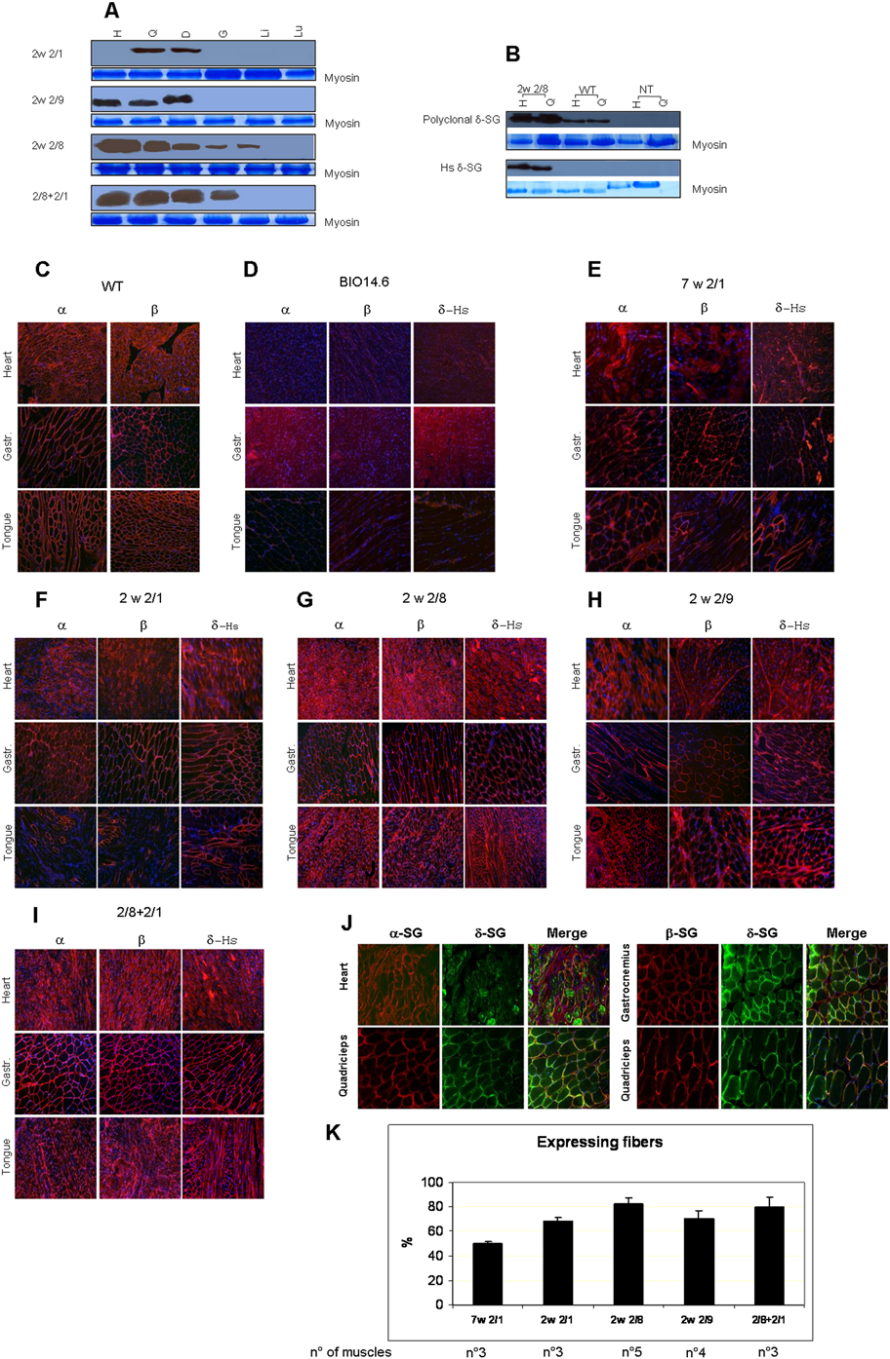
Expression and localization of the injected δ-SG. (A) Expression of δ-SG assessed by WB analysis in heart (H), quadriceps (Q), diaphragm (D), Gastrocnemius (G), Liver (Li) and lung (Lu) respectively of 2w 2/1, 2w 2/9, 2w 2/8 and 2/8+2/1 groups of hamsters. (B) WB performed using a polyclonal antibody against δ-SG and a monoclonal antibody against human δ-SG, respectively on heart and quadriceps of 2w 2/8, WT BIO14.6 hamsters. The expression and the localization, as well as the restoration of the SG complex was assessed through immunofluorescence (IF) staining respectively on heart, gastrocnemius and tongue of WT and BIO 14.6 not treated (C and D), compared with BIO14.6 hamsters that received alternative treatment protocols (E–I). IF were carried out by monoclonal antibodies against α-, β-, and human δ-SG respectively on 7w 2/1; 2w 2/1, 2w 2/8 and 2w 2/9; 2/8+2/1. The co-expression of SGs was determined with co-IF staining on several muscles of injected hamsters. Examples of co-IF staining on double injected hamsters (2/8+2/1) and 2w 2/9 hamsters are reported (J). Monoclonal antibody against alpha-SG, beta-SG or gamma-SG together with polyclonal antibody against delta-SG were used to test the co-expression of the proteins on the sarcolemma. They were stained respectively using secondary antibodies conjugated with Cy3 or FITC fluorochrome. On the left of the figure the merge shows the co-localization of δ-SG with α-, β- or γ-SG. Original magnification, 20×. (K) We calculated the percentage of fibers expressing δ-SG in the differently injected groups of hamsters. The percentages were evaluated estimating the number of expressing fibers in cryosections of gastrocnemius, quadriceps and tongue of each group of treated hamsters compared with BIO14.6. The number of the analysed hamsters are indicated in the figure. The bars represent the mean±the standard error of the analysed animals.

To test the expression of δ-SG, we analyzed tissue extracts by western blotting (Figure 2A), using a monoclonal Ab (NCL-d-SARC) against human δ-SG (Figure 1, stop signs). Since we delivered the human cDNA for the delta-sarcoglycan we can easily distinguish transgene protein from traces of endogenous hamster protein. The NCL-d-SARC cannot detect the hamster protein in WT animals, as it recognizes a human-specific N-terminal epitope (Figure 2B). We observed that the delta-sarcoglycan expression was faint in the 7w 2/1 hamsters (not shown). On the other hand, the expression of δ-SG was reproducible and evident in the muscles of 2w 2/8 hamsters (heart, gastrocnemius, quadriceps and diaphragm). The expression was variable in the skeletal muscles of 2w 2/1 and 2w 2/9 hamsters. In heart, the expression of δ-SG was weak or completely absent when we used AAV2/1. This result is in agreement with what has been observed with AAV2/6 that is very closely related to AAV2/1 [12]. In contrast, the heart of BIO14.6 expressed very high levels of δ-SG when we used serotype 2/8. Similar expression levels were seen in the 2/8+2/1 hamsters, but not in the 2/1+2/8 group. This result was evident up to 22 months of life (not shown). The δ-SG was expressed in the liver of some animals injected with serotype 2/8, but this expression did not cause any apparent hepatic abnormality at the histological level (not shown). No expression was observed in lung (Figure 2A), spleen, kidney and testis with any of the vectors used (not shown). The δ-SG expression level in the 2/8+2/1 and 2w 2/8 groups was in muscle and heart 3–4× higher than in the normal Syrian hamsters.

The recovery of the other components of the sarcoglycan complex was analyzed by indirect immunofluorescence staining. Antibodies against α-SG (NCL-a-SARC), β-SG (NCL-b-SARC), γ-SG (NCL-g-SARC) and human δ-SG (NCL-d-SARC) (Fig 2C–I) on 9-µ sections from heart, quadriceps, gastrocnemius, diaphragm, and tongue were used.

Double immunofluorescence staining with both rabbit anti δ-SG and mouse anti-α-SG or β-SG Ab showed a complete co-localization on muscle fibers (Figure 2J). The expression of the human δ-SG was constantly associated with the re-expression of the other sarcoglycan proteins belonging to the complex in all groups of animals. We also calculated the expressing fiber percentages in different tissues. The human δ-SG expressing fibers were on average 50.3% in the 7w 2/1 group, 68.2% in the 2w 2/1 hamsters, 82.9% in the 2w 2/8 hamsters, 70.4% in the 2w 2/9 hamsters and the 80.0% in the 2/8+2/1 hamsters (Figure 2K).

### Muscle pathology

**Figure 3:**
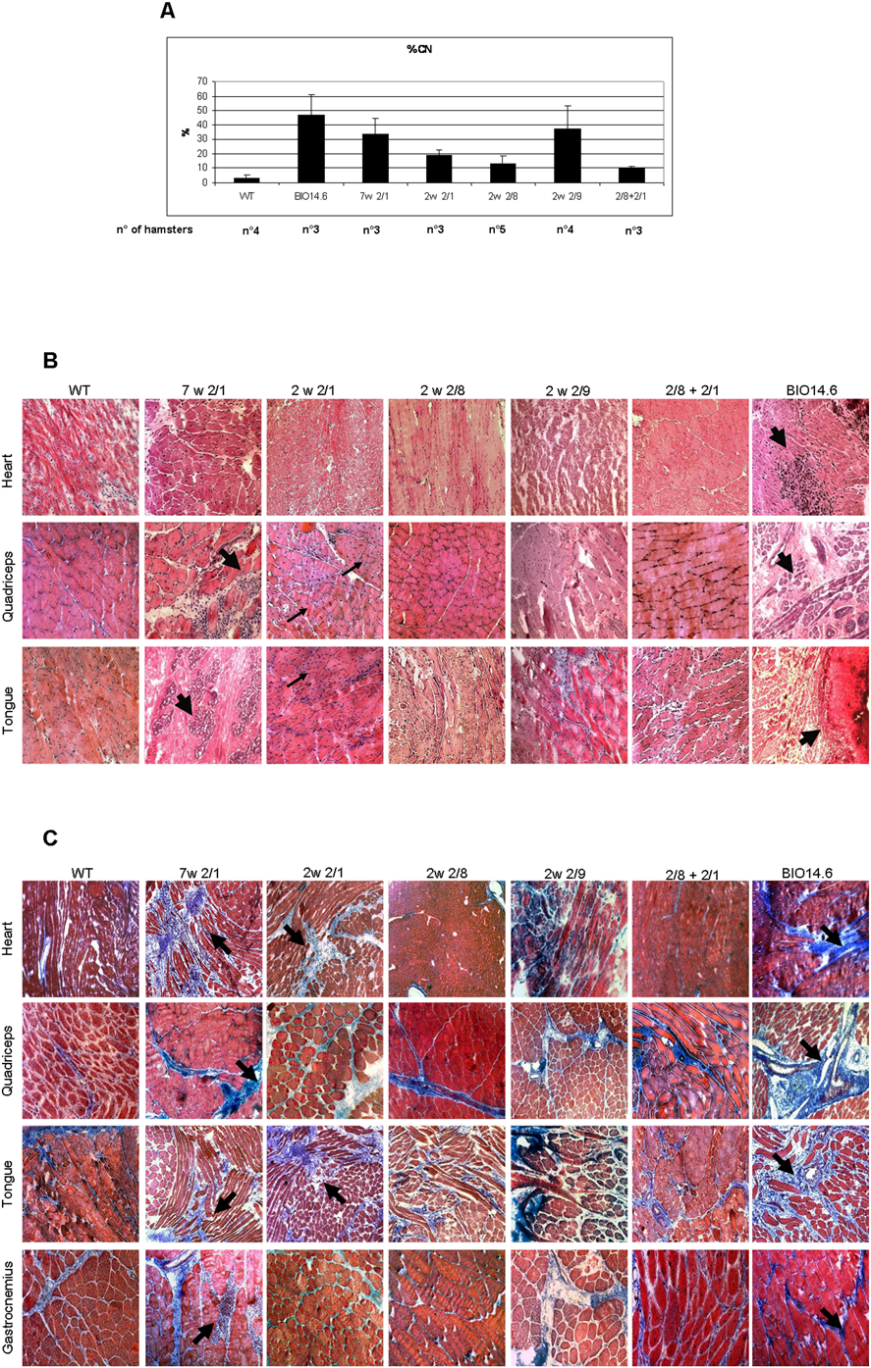
Gene delivery of delta-sarcoglycan through AAV vectors diminishes the percentage of centralized nuclei and the necrotic and fibrotic areas in heart and muscle. (A) Percentage of centralized nuclei respectively in 7w 2/1, 2w 2/1, 2w 2/8, 2w 2/9 and 2/8+2/1 groups of hamsters compared with BIO14.6 and WT hamsters. The bars represent the mean±the standard errors of the analysed animals. (B) Hematoxilin- Eosin staining on cryosections respectively of heart, quadriceps and tongue of wild type, treated and BIO14.6 hamsters. The black gross arrows indicate calcified and necrotic areas. The thin arrows underline the centralized nuclei. On the same groups of hamsters we performed Masson's trichrome staining on cryosection respectively of heart, quadriceps, tongue and gastrocnemius (C). The black gross arrows indicate fibrotic areas. All cryosections were analysed at 7 months of life. Original magnification, 20×.

To assess rescue of normal muscle structure, we first considered the percentage of centralized nuclei. In a normal hamster muscle, some rare nuclei can be observed at the center of cells (3%). These are regenerating fibers. In BIO14.6 hamsters, up to 60% of fibers have centralized nuclei. We counted the centralized nuclei of three different sections from quadriceps, gastrocnemius, diaphragm, and tongue. At least three hamsters for each group were evaluated at 1.5, 3 and 6 months respectively, comparing average values with age-matched WT and BIO14.6 hamsters We obtained an improvement in the 7w 2/1 (33.6%), 2w 2/1 (19.2%) and 2w 2/9 (37.6%) groups, compared with the untreated hamsters, in which the average percentage of centralized nuclei was 46.9% (Figure 3A). A much stronger reduction was observed in the 2w 2/8 (13.0%) and 2/8+2/1 (10.2%) groups. The percentage of centralized nuclei in 2w 2/8 and 2/8+2/1 groups were significatively different from the BIO14.6 hamsters *(p value = 0.000038)*.

We analysed the cardiac and skeletal muscle structure evaluating necrotic areas by hematoxylin-eosin staining (Figure 3B) and the presence of fibrosis by Masson's Trichrome procedure (Figure 3C). Since fibrotic areas appear in the course of the life, we evaluated the presence of fibrotic areas at 7 months of age for each group of hamsters. The 7w 2/1 hamsters showed large areas of necrosis and calcification, similar to the surviving untreated BIO14.6 at the same age. The best results were obtained in the 2w 2/8 and 2/8+2/1 animals, in which we noted the total absence of fibrotic and calcified areas and the lowest percentage of centralized nuclei. We found cardiomyocyte degeneration, necrosis and pronounced fibrosis in the untreated hamsters, but also in the 7w 2/1 hamsters.

### Non-invasive cardiac monitoring

**Figure 4:**
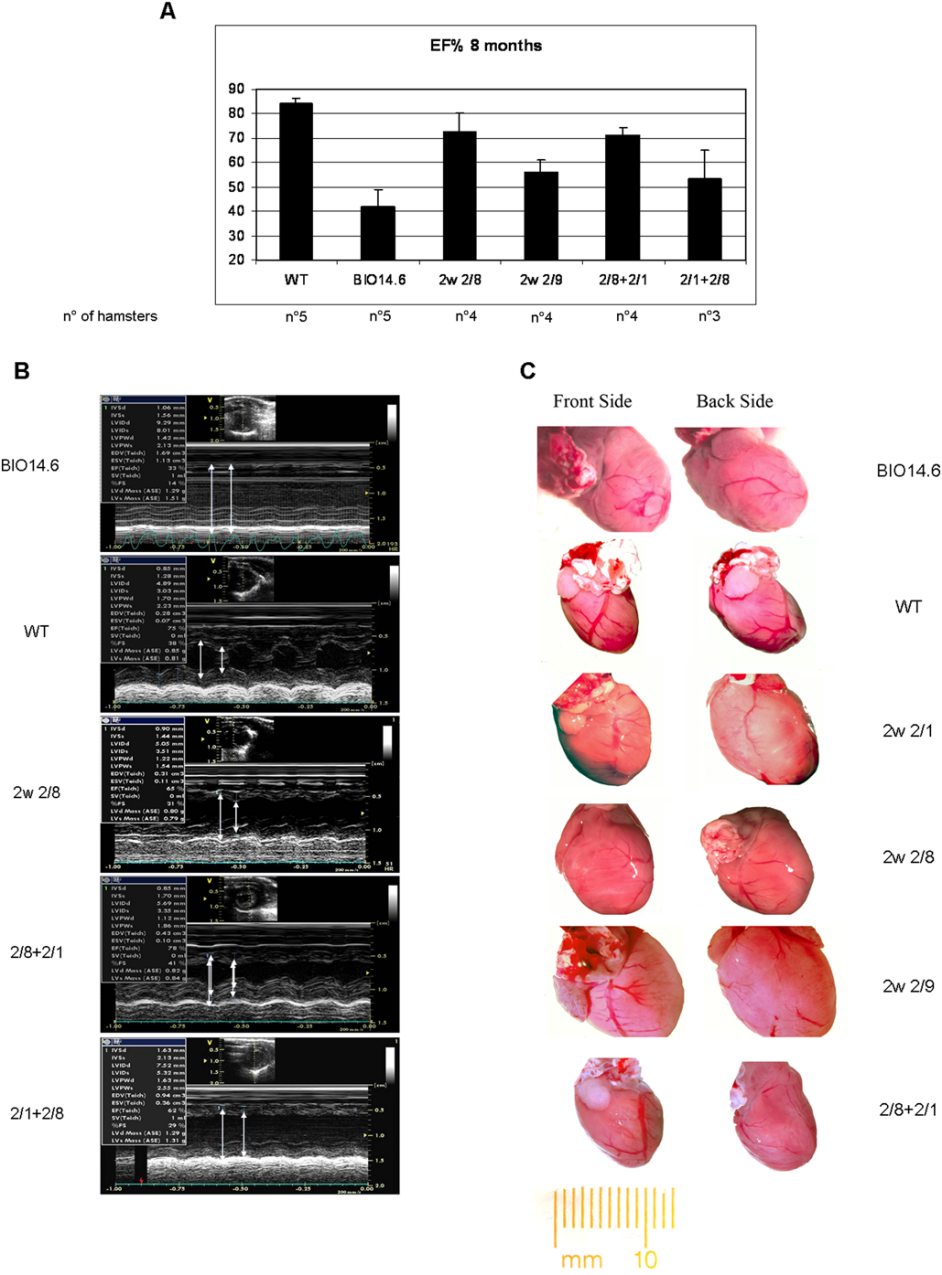
Morphologic and echocardiographic changes after gene delivery of delta-sarcoglycan in the differentially treated groups of hamsters. (A) Percentage of the ejection fraction in the 8-month-old BIO14.6 of differently treated hamsters, compared with the untreated BIO14.6 and the Wild Type F1B hamsters. The percentage calculated in the 2 w 2/8 and the 2/8+2/1 hamsters are significatively higher than the ones evaluated in the other groups (p-value: 2.8489e-006). The number of the analysed hamsters are indicated in the figure. The bars represent the mean±the standard error of the analysed animals. (B) Representative transthoracic left ventricular M-mode echocardiograms of 8-month-old BIO14.6 of differently treated hamsters, compared with the untreated BIO14.6 and the Wild Type F1B hamsters (C) The size of the heart of both 2w 2/8 and 2/8+2/1 hamsters is comparable to the size of WT hamsters' heart. Differently, the 7w 2/1 and the 2 w 2/9 groups show a very enlarged heart. The images were acquired at 7 months.

To monitor the major cardiac functional indexes, we used serial echocardiographies. We measured the left ventricle end-systolic and end-diastolic dimensions (LVD and LVS), the left ventricle posterior wall thickness, (indexes of cardiac hypertrophy). We calculated the ejection fraction (EF%) that is one of the most significative parameter to evaluate heart failure. The data were collected according to the timeline in Figure 1. At 8 months we detected significant improvement in all the groups of treated hamsters, and above all in the 2w 2/8 and 2/8+2/1 hamsters (Figure 4A and 4B).

The ejection fraction in both 2w 2/8 and AAV2/8+2/1 groups of hamsters was significatively different from the group of untreated BIO14.6 hamsters *(p value = 0.0000028)*.

We sacrificed some animals from each group at 7 months of age and removed the hearts. We observed enlarged gross heart morphology in the 7w 2/1 and 2w 2/1 animals, in a similar fashion with age-matched untreated hamsters. A very small increase in the heart size was in the 2w 2/8 hamsters, and a normal size in the 2/8+2/1 hamsters, similar to WT hamsters analysed at the same age (Figure 4C).

### Skeletal muscle function

To further confirm the skeletal muscle function recovery, animals for each group were tested by forced run on the rota-rod at a fixed speed (1.6 m/min for 45 minutes). At 6 months of age, BIO14.6 show reduced motility in the cage; in contrast, treated animals preserved spontaneous motility. On average, the WT hamsters did not fall off the rota-rod and never stopped running before the end of the test. The BIO14.6 animals could hardly run on the rota-rod, and stopped running more than 10 times during the exercise. The 2w 2/8 and 2/8+2/1 were indistinguishable from the WT (Movie S1). These data suggest that systemic delivery of human δ-SG by AAV vectors significatively ameliorated the cardiomyopathy and the muscular dystrophy in younger hamsters BIO14.6 than in the older ones and that the double injection can further improve the rescue of the phenotype in these animals.

### Long term study

**Figure 5:**
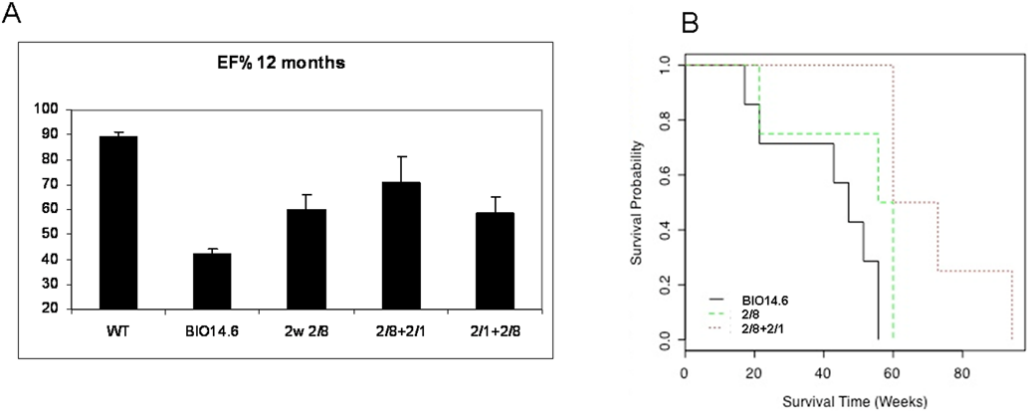
Long-term studies on surviving hamsters. (A) Histograms and standard errors of the mean of the ejection fraction (EF) percentage measured on the hamsters survived over 12 months in the groups under study. The two leftmost histograms, respectively the average EF value of the wild type F1B group (WT) and of the untreated BIO14.6, represent the two extremes of our measurements. The middle and the two rightmost histograms show the rescue on the FE due respectively to the treatments 2w 2/8, 2/8+2/1 and 2/1+2/8. It is easy to see that each of the three treatments is resulting in a gain of FE percentage and that in particular the 2/8+2/1 results to perform best. (B) Fleming-Harrington estimates of the survival curves respectively of the 2w 2/8, 2/8+2/1 and BIO14.6 groups of hamsters. The log-ratio test revealed, with a p-value of 0.002, that these three survival curves are significantly different.

To investigate whether the observed rescue was long-lasting, we also measured echocardiographic parameters in the surviving 12 month-old animals. We recalculated the ejection fraction (EF%). While all animals of the 2/8+2/1 group were still alive with sustained maintenance of good values of the ejection fraction (70.7% on average), in the group of 2w 2/8 two animals survived with an average ejection fraction of 59.8% (Figure 5A).

Finally, we calculated the survival curves respectively of the BIO14.6, 2w 2/8 and 2/8+2/1 animals and we found that percent survival as a function of time was significatively different (p<0.002) (Figure 5B).

## Discussion

We show that systemic treatment of muscular dystrophy and cardiomyopathy is feasible and efficacious with either a single or two sequential injections of AAV expressing delta sarcoglycan.

Muscle tissue represents about 40–50% of total body mass. In severe muscular dystrophies, respiratory failure [15] and/or heart failure [16] can be the causes of premature death. In addition, patients suffer from asymmetries in strength between reciprocal muscles that cause widespread joint and spine deformities requiring timely orthopaedic surgery [17]. Therefore, muscular dystrophies should be regarded as systemic disorders, and the rescue of single muscles can hardly obtain a significant therapeutic outcome.

An ideal therapy should be effective in 1) recovering balanced strength in all muscles; 2) preventing heart lesions; 3) improving respiratory function; 4) avoiding adverse immune reaction; 5) lasting along the entire lifespan. This compelled us to drive our research efforts towards testing the most effective systemic treatments.

AAV are among the most promising vectors for gene delivery *in vivo*, because they show widespread and sustained transgene expression after a single administration. AAV also showed efficient body-wide transduction capabilities when injected systemically using an intraperitoneal route in newborn animals. Novel serotypes have been introduced, each with peculiar tissue tropism. Muscle-targeting serotypes include AAV2/1, 2/5, 2/6, and 2/8 that show a transduction efficiency in mice up to 500 times greater than AAV2/2 [7], [18]–[22]. Previous reports evidenced the ability of AAV2/9 to transduce muscular tissues and in particular the myocardium, at 5- to 10-fold higher levels than AAV2/8 [14], [23]. One main limitation of AAV vectors was represented by its packaging ability that is restricted to 4.7 kb. This is a value compatible with the introduction of a small/medium-sized cDNA, such as delta-sarcoglycan that is 0.9 kb long. A recent paper demonstrated that vectors with AAV5 and AAV1 capsids can incorporate efficiently up to 8.9 kb of genome expanding the therapeutic potential of AAV vectors [24]. This could become suitable for dystrophin cDNA that can be further deleted to a compatible size without loss of function (mini-dystrophin) [25].

Sarcoglycan gene defects are an optimal model to test gene delivery, because the true function of the transgene can be followed through the recovery of the other sarcoglycan components [7], [26]–[28].

Zhu et al. obtained straightforward results using a single AAV8 injection in a related hamster (TO-2) that show a rapidly congestive cardiac phenotype [7]. This work constitutes the basis to test alternative AAV delivery strategies for long-term efficacy. To optimize gene delivery protocols, reproducibility is a “must”, since animals may be dissimilar in disease course and/or in disease rescue [29]. We chose the male BIO14.6 Syrian hamster model for three main reasons: a) it is a genetically homogeneous strain, since it has a 35-year long inbreeding story by Bio-Breeders [30]; b) it has a reproducible schedule of both cardiomyopathy and muscular dystrophy, documented by a very large number of studies; c) the disease course is severe and the lifespan is shortened, but not so much, to follow long-term disease progression. Heart disease is similar to the dilated cardiomyopathy of human muscular dystrophies [31], [32] and can be followed during the entire lifespan of the animal by serial echocardiographies.

The initial approach was to compare different protocols, to establish the best methodology for systemic delivery of δ-SG. The parameters tested were: the route of injection, the age and the number of injections, and the AAV serotypes.

1. The route of injection.

We injected adult hamsters systemically into the jugular vein, whilst the young animals were injected intra-peritoneally, assuming this way as systemic according to Xiao [7]. We observed that the serotype 2/8 was capable of delivering δ-SG in muscle and heart in both cases while the serotype 2/1 did not reach the heart efficiently.

2. The age of injection.

Previous studies have assessed the efficiency of injecting AAV vectors expressing δ-SG under the control of the muscle specific synthetic promoter SP instead of the CMV promoter in both young (10 days old) and adult hamsters [33]. We administered the same dosage of AAV2/1 expressing the δ-SG under the control of the CMV promoter both into adult hamsters and into newborn ones. When we treated newborn hamsters, we observed a dramatic change for each histological parameter considered, i.e. the percentage of fibers expressing δ-SG, the percentage of centralized nuclei, and the absence of large fibrotic or necrotic areas. This observation suggests that the injection in younger hamsters is more efficient probably because, in adult animals, necrotic and fibrotic areas may reduce the transduction into the muscle.

3. AAV serotypes.

We tested different serotypes, because there are conflicting results on the transduction ability of AAV serotypes to infect cardiac and muscular tissues (and these are mainly referred to mice) [7], [11], [12], [23] We used AAV2/1, AAV2/8, and AAV2/9, also in combination, performing two injections at different times into the same hamsters. We observed the best transduction efficiency in the hamster treated at 2 weeks with the AAV2/8 serotype, which showed high percentage of expressing fibers, low percentage of centralized nuclei, absence of necrotic or fibrotic areas and a general improvement in each echocardiographic parameter. Longer-lasting results were obtained with the double injection 2/8+2/1, since the survival curve was significatively different from 2w 2/8 and untreated animals. This suggests that the double treatment could maintain a longer expression of delta-sarcoglycan. Interestingly, one of the BIO14.6 hamsters receiving the double injection 2/8+2/1 lived up to 22 months (+100% of expected lifespan). Furthermore, we observed that all the double injected 2/8+2/1 hamsters were still alive at 16 months, showing 70% of ejection fraction. This percentage declined to 59.8% and 58.5% respectively, in two surviving 2w 2/8 and 2/1+2/8 animals while the 2w 2/9 and the untreated hamsters at the same age were dead. We also confirmed that the serotype 2/8 is the most efficient of those tested for intraperitoneal delivery into newborn hamsters [13]. We hypothesized that, after the first neonatal injection, the degeneration of muscle to fibrocalcific/fibroadipous tissue was prevented, thus a late injection with a different serotype could result in a transduction of the surviving muscle and boost the expression of the delta-sarcoglycan. This explains why the first injection at 7w of AAV2/1 was less effective than that performed at 5 months in 2w 2/8 hamsters.

Our results with serotype 2/9 are unsatisfactory and it was in contrast with the previous observations on the ability of AAV2/9 in mice to transcend vasculature and transduce myocardium [14]. We showed that the 2w 2/9 hamsters showed much weaker transduction than the 2w 2/8 animals and this result was confirmed using different AAV preparations. One possible explanation could be related to the intraperitoneal route of administration [13]

To avoid cardiomyopathy it is necessary to have a sustained and homogeneous distribution of the rescued fibers. This situation is dissimilar from what happens for the skeletal muscle where levels of about 30% of dystrophin are sufficient to avoid disease [34]. Interestingly, heterozygous carriers of dystrophin defects have no skeletal muscle disease, but they show dilated cardiomyopathy [35]. This is in contrast with the results of Bostick on mdx mice [36]. It is possible that the penetrance of cardiac phenotype in mice is lower than in human and hamsters. BIO14.6 hamsters having less than 70% of fibers expressing delta-sarcoglycan developed cardiomyopathy, even if the total amount of protein measured by western blot analysis was in the normal range or 3–4-fold above the normal range. This elevated expression did not cause any problems for heart or muscle in contrast to what was previously observed in transgenic mice bearing the δ-SG S151A allele [37].

This underscores the importance that patients receive homogeneously the missing protein; otherwise the infection will be ineffective to rescue the phenotype.

The advantage of testing the efficacy of a second injection using a second serotype is related to the possibility both of improving the survival and extending the therapeutic window as was observed in our experiments. Patients treated with single AAV may have a second chance using a different serotype. This could be relevant considering that ongoing human trials with proof-of-principle intramuscular injections can produce life-long immunization against AAV.

In conclusion we showed long term biochemical, morphological and functional improvement in a rodent model of sarcoglycanopathy following AAV mediated body-wide muscle transduction. This has important implication for therapies of life treatment and incurable muscular dystrophies.

## Materials and Methods

### AAV vector construction and production

The human δ-SG gene was cloned into the plasmid pAAV-2.1CMV-EGFP. Recombinant AAV vector containing human delta-sarcoglycan cDNA, driven by the cytomegalovirus (CMV) promoter, were constructed by standard cloning protocols and were packaged into AAV2/1, 2/8 and 2/9 serotypes. The resulting pAAV2.1-CMV-delta-SG were transfected in sub-confluent 293 cells along with the pAd-Helper and the pack 2/8, pack 2/1 or pack 2/9 packaging plasmids, as described previously [38]. The recombinant AAV2/8, 2/1 or 2/9 vectors were purified by two rounds of CsCl, as described previously. Vector titers, expressed as genome copies (GC/ml), were assessed by real-time PCR (GeneAmp 7000 Applied Biosystem), as described previously [39].

### Animals and vector administration

All procedures on wild-type F1B and dystrophic BIO14.6 hamsters (Bio Breeders, Fitchburg, Mass) have been approved by the relevant Local Committees for “Good Animal experimental Activities”.

### Western Blotting

Muscle or other tissues were homogenized and lysed in a lysis assay buffer (Urea 8M, SDS 4%, 125 mM Tris HCl pH 6.8). The samples were separated on sodium dodecyl sulfate-10% polyacrylamide gel electrophoresis and transferred to nitrocellulose membrane. After blocking in 10% non-fat dry milk in Phosphate-buffered saline (PBS-1X) buffer (10 mM Tris-HCl, 200 mM NaCl, 0.05% NP 40, 0.05% TWEEN 20) for 1 h, the membranes were incubated with primary antibodies in PBS-1X at room temperature for 2 h. The monoclonal antibody recognizing a human epitope of δ-SG was used in this experiment with a 1∶25 dilution. Following primary antibody incubation and rinses, the membranes were incubated with the secondary antibody, goat anti-mouse immunoglobulin conjugated with horseradish peroxidase (Sigma), with 1∶15,000 dilution in 0.5% dry milk and PBS-1X buffer. After a 1-h antibody incubation and three washes with PBS-1X buffer, the δ-SG protein band was visualized with a chemiluminescence reagent (Supersignal, WestPico, Pierce) and exposed to X-ray film.

### Histology

Muscle or other tissues were collected at 2, 3 or 6 months after vector injection. Tissues samples were processed by cryosections at 7- to 10- µm thickness.

### Hematoxylin-Eosin staining

Cryosections of muscular tissues were fixed in 4% PFA, then washed in PBS-1X and then they were stained in hematoxylin for 4 minutes and eosin for 6 minutes. Then the cryosections were dried in ethanol and at the end they were fixed in xylene and mounted with EUKITT mounting (O. Kindler GmbH & CO).

### Masson's trichrome staining

Infiltrations of fibrotic tissue respectively in heart, gastrocnemius, quadriceps and tongue were observed by Masson's trichrome staining. (9 µm of thickness). Cryosections of muscular tissues were fixed in Bouin's Solution at 56°C for 15 minutes, cooled and washed in running tap water to remove yellow colour from section. They were stained in Working Weigert's Iron Hematoxilin Solution for 5 minutes, washed in running tap water for 5 minutes and stained in Biebrich Scarlet- Acid Fucsin for 5 minutes, then rinsed in deionized water, placed in Working Phosphotungstic\Phosphomolybdic acid solution for 5 minutes, stained in Aniline Blue solution for 5 minutes and in acid acetic 1% for 2 minutes. The slides were mounted with EUKITT mounting (O. Kindler GmbH & CO).

### Immunofluorescence

For immunofluorescence staining, the unfixed muscle cryosections were immediately blocked in 10% goat serum and phosphate-buffered saline (PBS-1X) at room temperature for 1 h. Monoclonal antibodies (NCL-a-SARC, NCL-b-SARC, NCL-g-SARC and NCL-d-SARC), respectively against α-SG, β-SG, γ-SG and δ-SG were used (Novocastra Laboratories). α-SG, β-SG and γ-SG were diluted at 1∶100; the monoclonal antibody against δ-SG was diluted 1∶25; the polyclonal antibody to δ-SG was prepared in our laboratory^6^. It was diluted 1∶1000 in 10% goat serum-PBS-1X and incubated with the cryosections for 1 h and 30′ at room temperature. After three washes, the sections were incubated with Cy-3-labeled antimouse or antirabbit secondary antibodies respectively at 1∶300 and 1∶400 dilution in 10% goat serum-PBS-1X (Jackson Immuno Research Laboratories). After three washes, the samples were mounted in Vectashield mounting with DAPI (Vector Laboratories). All sections were acquired under a Zeiss Axioplan fluorescence microscope, using Axio Vision 4.5 software at a magnification of 20×.

### Echocardiographic Study

All hamsters received baseline echocardiograms under controlled anesthesia and spontaneous respiration, using a Vivid 7 (GE VingMed, Horten, Norway) with a linear 13 MHz probe (i13L). The anterior chest hair was removed using a shaver and the hamsters were positioned in left lateral decubitus on a wooden bench. Recordings were made under continuous ECG monitoring by fixing the electrodes on the limbs.

### Grey scale imaging

Grey scale images were recorded in a parasternal short axis view at a depth of 2 cm. M-Mode tracings were recorded at the level of the papillary muscles at a speed of 200 mm/s. Left ventricular (LV) dimensions were measured from three consecutive cardiac cycles on the M-Mode tracings. The Teichholz formula was used to calculate LV volumes: Pi*D3/6a (D ¼ diameter of the ventricle in short axis view; a ¼ ellipticity factor. An ellipticity factor of 1/3 was used, assuming that L ¼ 3D (L ¼ total length of the ellipse). LVEF was calculated as LV end-diastolic volume e LV end-systolic volume/LV end-diastolic volume and expressed in %.

### Statistics

We performed a one way analysis of variance (ANOVA) for testing the effects of the grouping variable on the mean respectively of the Ejection Fraction (EF) and of the observed NC percentage (NC). We used a Fleming-Harrington test for the analysis of the survival.

We performed a posterior multiple comparison test at level 0.05, with a Bonferroni adjustment for multiplicity.

## Supporting Information

Movie S1 Hamsters respectively not treated (lane 1), treated at 2 weeks with serotype 2/8 (lane 2) and double treated, 2/8+2/1 (lane 3), after 40 min of exercise at a constant speed.
(12.32 MB AVI)

## References

[1] Matsumura K, Tome FM, Collin H, Azibi K, Chaouch M (1992). Deficiency of the 50 K dystrophin-associated glycoprotein in severe childhood autosomal recessive muscular dystrophy.. Nature.

[2] Campbell KP, Kahl SD (1989). Association of dystrophin and an integral membrane glycoprotein.. Nature.

[3] Ozawa E, Mizuno Y, Hagiwara Y, Sasaoka T, Yoshida M (2005). Molecular and cell biology of the sarcoglycan complex.. Muscle & Nerve.

[4] Nigro V, de Sa Moreira E, Piluso G, Vainzof M, Belsito A (1996). Autosomal recessive limb-girdle muscular dystrophy, LGMD2F, is caused by a mutation in the delta-sarcoglycan gene.. Nat Genet.

[5] Homburger F, Baker JR, Nixon CW, Wilgram G (1962). New hereditary disease of Syrian hamsters. Primary, generalized polymyopathy and cardiac necrosis.. Arch Intern Med.

[6] Nigro V, Okazaki Y, Belsito A, Piluso G, Matsuda Y (1997). Identification of the Syrian hamster cardiomyopathy gene.. Hum Mol Genet.

[7] Zhu T, Zhou L, Mori S, Wang Z, McTiernan CF (2005). Sustained whole-body functional rescue in congestive heart failure and muscular dystrophy hamsters by systemic gene transfer.. Circulation.

[8] Chamberlain JS, Metzger J, Reyes M, Townsend D, Faulkner JA (2007). Dystrophin-deficient mdx mice display a reduced life span and are susceptible to spontaneous rhabdomyosarcoma.. Faseb J.

[9] Muntoni F, Cau M, Ganau A, Congiu R, Arvedi G (1993). Brief report: deletion of the dystrophin muscle-promoter region associated with X-linked dilated cardiomyopathy.. N Engl J Med.

[10] Choi VW, McCarty DM, Samulski RJ (2005). AAV hybrid serotypes: improved vectors for gene delivery.. Curr Gene Ther.

[11] Jiang H, Pierce GF, Ozelo MC, de Paula EV, Vargas JA (2006). Evidence of multiyear factor IX expression by AAV-mediated gene transfer to skeletal muscle in an individual with severe hemophilia B.. Mol Ther.

[12] Gregorevic P, Blankinship MJ, Allen JM, Crawford RW, Meuse L (2004). Systemic delivery of genes to striated muscles using adeno-associated viral vectors.. Nat Med.

[13] Wang Z, Zhu T, Qiao C, Zhou L, Wang B (2005). Adeno-associated virus serotype 8 efficiently delivers genes to muscle and heart.. Nat Biotechnol.

[14] Pacak CA, Mah CS, Thattaliyath BD, Conlon TJ, Lewis MA (2006). Recombinant adeno-associated virus serotype 9 leads to preferential cardiac transduction in vivo.. Circ Res.

[15] Simonds AK (2006). Recent advances in respiratory care for neuromuscular disease.. Chest.

[16] Nigro G, Comi LI, Politano L, Bain RJ (1990). The incidence and evolution of cardiomyopathy in Duchenne muscular dystrophy.. Int J Cardiol.

[17] Do T (2002). Orthopedic management of the muscular dystrophies.. Curr Opin Pediatr.

[18] Chao H, Liu Y, Rabinowitz J, Li C, Samulski RJ, Walsh CE (2000). Several log increase in therapeutic transgene delivery by distinct adeno-associated viral serotype vectors.. Mol Ther.

[19] Duan D, Yan Z, Yue Y, Ding W, Engelhardt JF (2001). Enhancement of muscle gene delivery with pseudotyped adeno-associated virus type 5 correlates with myoblast differentiation.. J Virol.

[20] Scott JM, Li S, Harper SQ, Welikson R, Bourque D (2002). Viral vectors for gene transfer of micro-, mini-, or full-length dystrophin.. Neuromuscul Disord.

[21] Blankinship MJ, Gregorevic P, Allen JM, Harper SQ, Harper H (2004). Efficient transduction of skeletal muscle using vectors based on adeno-associated virus serotype 6.. Mol Ther.

[22] Chao H, Monahan PE, Liu Y, Samulski RJ, Walsh CE (2001). Sustained and complete phenotype correction of hemophilia B mice following intramuscular injection of AAV1 serotype vectors.. Mol Ther.

[23] Inagaki K, Fuess S, Storm TA, Gibson GA, McTiernan CF (2006). Robust systemic transduction with AAV9 vectors in mice: efficient global cardiac gene transfer superior to that of AAV8.. Mol Ther.

[24] Allocca M, Doria M, Petrillo M, Colella P, Garcia-Hoyos M (2008). Serotype-dependent packaging of large genes in adeno-associated viral vectors results in effective gene delivery in mice.. J Clin Invest.

[25] Ragot T, Vincent N, Chafey P, Vigne E, Gilgenkrantz H (1993). Efficient adenovirus-mediated transfer of a human minidystrophin gene to skeletal muscle of mdx mice.. Nature.

[26] Holt KH, Lim LE, Straub V, Venzke DP, Duclos F (1998). Functional rescue of the sarcoglycan complex in the BIO 14.6 hamster using delta-sarcoglycan gene transfer.. Mol Cell.

[27] Li J, Wang D, Qian S, Chen Z, Zhu T, Xiao X (2003). Efficient and long-term intracardiac gene transfer in delta-sarcoglycan-deficiency hamster by adeno-associated virus-2 vectors.. Gene Ther.

[28] Ikeda Y, Gu Y, Iwanaga Y, Hoshijima M, Oh SS (2002). Restoration of deficient membrane proteins in the cardiomyopathic hamster by in vivo cardiac gene transfer.. Circulation.

[29] Kobuke K, Piccolo F, Garringer KW, Moore SA, Sweezer E (2008). A Common Disease-Associated Missense Mutation in Alpha-Sarcoglycan Fails to Cause Muscular Dystrophy in Mice.. Hum Mol Genet.

[30] Homburger F (1979). Myopathy of hamster dystrophy: history and morphologic aspects.. Ann N Y Acad Sci.

[31] Cohen N, Muntoni F (2004). Multiple pathogenetic mechanisms in X linked dilated cardiomyopathy.. Heart.

[32] Muntoni F (2003). Cardiomyopathy in muscular dystrophies.. Curr Opin Neurol.

[33] Li X, Eastman EM, Schwartz RJ, Draghia-Akli R (1999). Synthetic muscle promoters: activities exceeding naturally occurring regulatory sequences.. Nat Biotechnol.

[34] Neri M, Torelli S, Brown S, Ugo I, Sabatelli P (2007). Dystrophin levels as low as 30% are sufficient to avoid muscular dystrophy in the human.. Neuromuscul Disord.

[35] Politano L, Nigro V, Nigro G, Petretta VR, Passamano L (1996). Development of cardiomyopathy in female carriers of Duchenne and Becker muscular dystrophies.. JAMA.

[36] Bostick B, Yue Y, Long C, Duan D (2008). Prevention of dystrophin-deficient cardiomyopathy in twenty-one-month-old carrier mice by mosaic dystrophin expression or complementary dystrophin/utrophin expression.. Circ Res.

[37] Heydemann A, Demonbreun A, Hadhazy M, Earley JU, McNally EM (2007). Nuclear sequestration of delta-sarcoglycan disrupts the nuclear localization of lamin A/C and emerin in cardiomyocytes.. Hum Mol Genet.

[38] Gao GP, Alvira MR, Wang L, Calcedo R, Johnston J, Wilson JM (2002). Novel adeno-associated viruses from rhesus monkeys as vectors for human gene therapy.. Proc Natl Acad Sci U S A.

[39] Gao G, Qu G, Burnham MS, Huang J, Chirmule N (2000). Purification of recombinant adeno-associated virus vectors by column chromatography and its performance in vivo.. Hum Gene Ther.

